# Ambient Assisted Living: Scoping Review of Artificial Intelligence Models, Domains, Technology, and Concerns

**DOI:** 10.2196/36553

**Published:** 2022-11-04

**Authors:** Mladjan Jovanovic, Goran Mitrov, Eftim Zdravevski, Petre Lameski, Sara Colantonio, Martin Kampel, Hilda Tellioglu, Francisco Florez-Revuelta

**Affiliations:** 1 Department of Computer Science, Singidunum University Belgrade Serbia; 2 Faculty of Computer Science and Engineering, University Saints Cyril and Methodius Skopje North Macedonia; 3 Signals & Images Lab, Institute of Information Science and Technologies, National Research Council of Italy Pisa Italy; 4 Faculty of Informatics, Vienna University of Technology Vienna Austria; 5 Department of Computing Technology, University of Alicante Alicante Spain

**Keywords:** ambient assisted living, AAL, assisted living, active living, digital health, digital well-being, automated learning approach, artificial intelligence algorithms, human-centered AI, review, implications, artificial intelligence, mobile phone

## Abstract

**Background:**

Ambient assisted living (AAL) is a common name for various artificial intelligence (AI)—infused applications and platforms that support their users in need in multiple activities, from health to daily living. These systems use different approaches to learn about their users and make automated decisions, known as AI models, for personalizing their services and increasing outcomes. Given the numerous systems developed and deployed for people with different needs, health conditions, and dispositions toward the technology, it is critical to obtain clear and comprehensive insights concerning AI models used, along with their domains, technology, and concerns, to identify promising directions for future work.

**Objective:**

This study aimed to provide a scoping review of the literature on AI models in AAL. In particular, we analyzed specific AI models used in AАL systems, the target domains of the models, the technology using the models, and the major concerns from the end-user perspective. Our goal was to consolidate research on this topic and inform end users, health care professionals and providers, researchers, and practitioners in developing, deploying, and evaluating future intelligent AAL systems.

**Methods:**

This study was conducted as a scoping review to identify, analyze, and extract the relevant literature. It used a natural language processing toolkit to retrieve the article corpus for an efficient and comprehensive automated literature search. Relevant articles were then extracted from the corpus and analyzed manually. This review included 5 digital libraries: IEEE, PubMed, Springer, Elsevier, and MDPI.

**Results:**

We included a total of 108 articles. The annual distribution of relevant articles showed a growing trend for all categories from January 2010 to July 2022. The AI models mainly used unsupervised and semisupervised approaches. The leading models are deep learning, natural language processing, instance-based learning, and clustering. Activity assistance and recognition were the most common target domains of the models. Ambient sensing, mobile technology, and robotic devices mainly implemented the models. Older adults were the primary beneficiaries, followed by patients and frail persons of various ages. Availability was a top beneficiary concern.

**Conclusions:**

This study presents the analytical evidence of AI models in AAL and their domains, technologies, beneficiaries, and concerns. Future research on intelligent AAL should involve health care professionals and caregivers as designers and users, comply with health-related regulations, improve transparency and privacy, integrate with health care technological infrastructure, explain their decisions to the users, and establish evaluation metrics and design guidelines.

**Trial Registration:**

PROSPERO (International Prospective Register of Systematic Reviews) CRD42022347590; https://www.crd.york.ac.uk/prospero/display_record.php?ID=CRD42022347590

## Introduction

### Background

Ambient assisted living (AAL) is an umbrella term describing a general approach to technology design to construct safe environments around assisted users and help them maintain independent living [1]. Over time, it has focused mainly on older adults (people aged >65 years) as target users.

Developing technology for this group is an increasingly important design challenge because of specific deficits in later life [2]. Beyond usability, there is an increased emphasis on designing technology for older adults that will enable them not merely to satisfy their needs but also to transform their mental and physical health and well-being [3,4]. This challenge is particularly significant because older people are becoming the largest demographic group. In 2020, more than one-fifth (20.6%) of the population in the European Union was aged ≥65 years [5], and by 2030, an estimated 16.6% of the world’s population will be aged ≥60 years [6].

Over the last decade, many technological devices have been developed to support an active lifestyle as people age, concerning health promotion [4,7,8]. Health promotion refers to “the process of empowering people to increase control over their health and its determinants through health literacy efforts and multisectoral action to increase healthy behaviors” [9]. Concerning technology design, the objective is to find cost-effective solutions to help independent living and provide health care and well-being [10]. A comprehensive analysis of information and communication technology research and development revealed the common goals of these technologies as the provision of health, accessibility, and safety [11,12]. Many technologies seek to assist older adults with everyday activities [4,11,13-15].

To support disability-free and independent living and the well-being of older users, AAL systems use automated decision-making mechanisms that integrate, analyze, and interpret complex multimodal and multidevice information [7]. These systems have focused on 2 general scenarios of automated decision-making involving older users—*health monitoring* and *activity recognition* [8,16,17].

Different *monitoring* contexts were targeted by a variety of technological systems, ranging from monitoring systems for fall prevention using wearable and ambient sensing technology [18] and social robots for the well-being of people with dementia and mild cognitive impairments [13] to games for leisure and user engagement during therapy and rehabilitation [14].

Robotic technologies have been widely exploited as tools to support health monitoring and mobility capacities, such as strength, balance, and range of motion [15], or as companions [19] to assist older adults in daily and social activities at home. The former may be nonsocial robots, whereas the latter are social robots with the primary goal of offering companionship.

Remote telepresence robots have been successfully used to support the autonomy of older adults in doing daily activities at home. Giraff is a telepresence robot that uses a video interface to allow caregivers and relatives to visit older people in their homes remotely [20]. It runs implicit data collection (blood pressure, body temperature, movement, and fall) and then analyzes the data to alert the caregivers for emergencies. Similarly, Matilda is a social robot with human attributes (such as baby-face–like appearance, human voices, gestures, and body movements) that can recognize voices and faces and perform activities such as playing music, dancing, and playing card games [21].

Although biological aging cannot be stopped, regular exercise can minimize its physiological effects, increase life satisfaction, and prolong the decline in functional abilities in older adults [22]. Studies on the favorite activities of older adults show the prominence of physical activities such as walking, jogging, and outdoor maintenance [23]. Specific technologies, such as exergames [10] or web-based exercises and activities [24,25], have motivated, sustained, and monitored physical and social activities at older adults’ homes. Coupling with the features from theories of human behavior, such as goal setting, self-monitoring, achievements, and personalized feedback and progression, has been associated with the higher effectiveness of these applications for older adults (ie, increased engagement in physical activities and associated health outcomes) [26].

In the context of AAL, *activity recognition* concerns tracking the daily behavior of older and frail people. It can detect falls and recognize activities of daily living (ADL), which are crucial for identifying complex patterns associated with the development of specific diseases. Zdravevski et al [27] suggested an automated approach for analyzing multivariate time series originating from various sensors and facilitating the robust classification of daily activities.

*Wearable* [28] and *mobile* technologies [29,30] have been used for implicit data collection and analysis to recognize older adults’ activities for tracking their health and detecting emergencies.

From a technical perspective, the energy efficiency of wearable technologies appears to be the primary constraint for continuous measurement and activity recognition [28]. It further affects the provision of timely and informative feedback and recommendations for the users. The major user-related concerns are privacy and acceptance [28] due to unclear use cases and difficulties in device pairing with a smartphone for older adults. A more stable commitment to wearables requires use cases with apparent benefits and reduced effort of use for older adults.

Mobile technologies represent a versatile source for older adults’ health and activity data collection [29,30]. They facilitate home care and self-management of the health and well-being of older adults. These applications implement various services based on target activity and health recognition features to support health care and independent living (ie, reminders, companionship, or recommendation of favorite activities or treatments). However, significant obstacles to using mobile technologies in practice include privacy [30] and technological literacy and usability of touch screen interaction styles [31].

AAL technologies use a variety of artificial intelligence (AI) models in learning about their users’ habits and health conditions to provide adequate services with automated decision-making. Table 1 shows the common AI classification, whereas Table 2 summarizes existing AI models concerning their learning and decision-making techniques and the problems they address (with corresponding algorithms) [32-34]. We separated the classification and models because multiple models can belong to the same class. Conversely, some models can implement different classes (ie, clustering can be done in both supervised and unsupervised manners).

**Table 1:** The artificial intelligence classification as common learning approaches [32-34].

Name	Description	Problem or algorithm
Supervised learning	Input (training) data or examples are labeled with known output values. The model uses the data in a training process to make predictions, and the predictions are corrected when they are false. The process runs until the model achieves a required level of the prediction accuracy.	Classification and regression
Unsupervised learning	Input data are not labeled, and output values are unknown. Instead, the model is trained by removing structures from the input data to extract general rules, reduce redundancy, or organize data by similarity.	Clustering, dimensionality reduction, and association rule learning
Semisupervised learning	Input data contains labeled and unlabeled examples. The model learns the structures to organize the data to create predictions. It models the unlabeled data.	Classification and regression
Reinforcement learning	The model rewards desired behaviors and eliminates undesired ones. It is represented by a learning agent (process) that perceives and interprets its environment, takes actions, and learns through trial and error.	Markov Decision Process, Q learning, and Monte Carlo methods

**Table 2:** The summary of artificial intelligence models [32-34].

Model	Learning technique	Problem or algorithm
Regression learning	Models a relationship between input and output data (or variables). The relation is iteratively refined by measuring errors in the model’s predictions.	Variations such as linear and logistic regression
Instance-based learning	Models a decision based on instances of input data that are considered relevant or necessary. Creates a database of reference examples used to compare with new data to find optimal matches using similarity metrics to make a decision.	K-nearest neighbor and support vector machines
Regularization learning	The extension or modification of another model (eg, regression learning) in a way that reduces the complexity of the model by converting it into a simpler form.	Ridge regression and elastic net regression
Decision tree learning	Models a decision based on the values of the input data attributes. It follows a tree structure in making a decision for given input data.	Classification and regression trees and conditional decision tree
Bayesian learning	The models use Bayes’ theorem to solve problems of classification and regression.	Naïve Bayes and Gaussian naïve Bayes
Clustering learning	The model organizes the input data into groups (or clusters) where group membership or commonality criteria are taken or derived from the data (eg, centroid based or hierarchical).	K-means, K-medians, and hierarchical clustering
Association rule learning	The model discovers associations in input data to make a decision. It extracts rules that describe relationships between observed variables in input data.	A priori algorithm and Eclat algorithm
Artificial neural network	The model is driven by the structure and function of the human neural networks. Represents a class of pattern matching models and their commonly used variations for regression and classification problems.	Perceptron, multilayer perceptrons, and back propagation
Deep learning	Special category of large and complex neural networks for handling vast amounts of labeled input data, including text, images, audio, and video.	Convolutional neural network, recurrent neural networks, and long short-term memory networks
Dimensionality reduction learning	The model analyzes the input structure in the data to represent and describe the data with less information. The simplified data can be visualized and used by other learning methods.	Principal component analysis, principal component regression, and linear discriminant analysis
Ensemble learning	Multiple models that are independently trained, where individual predictions are combined to make the final prediction. The models are combined owing to their weaknesses in making the desired prediction.	Boosting, random forest, AdaBoost, and weighted average (blending)
Natural language processing	Specific for conversational artificial intelligence and includes natural language understanding, dialog management, and natural language generation.	Rule-based algorithms, statistics, neural networks, and deep learning

### Goal of the Study

This study investigated the AI models of existing AAL technologies to support independent living. The quality of the models’ decision-making can benefit positive behavior change to maintain an active and healthy lifestyle for older adults and other user groups in need of assistance. This is critical for preventing functional decline and supporting health treatments. Our work aimed to identify the positive aspects and gaps in research and practice to provide implications for future AAL systems.

This scoped analysis focused on the following research questions (RQs):

1. RQ1: What AI models are implemented in AAL systems?
  First, we identified, described, and systematized AI classifications and models in the current landscape of AAL systems. For this purpose, we extracted common terminology to describe current AI models and AAL.
2. RQ2: What are the domains of the models?
  Second, we described existing target domains with their concrete activities to propose suitable application strategies that reinforce positive aspects and highlight critical parts in which further research is necessary.
3. RQ3: What technologies are using the models?
  Third, we investigated different technologies using AI models to consolidate and provide design and development guidelines for intelligent AAL systems.
4. RQ4: What are the significant concerns regarding the models from an end-user perspective?
  Finally, we examined end-user groups and their perceptions of AAL system use to indicate specific requirements that the systems should meet or improve.

This study reviews AI models in AAL concerning their domains, technologies, and concerns published in the literature covering 2010 to 2022. The findings are intended for health and care professionals, researchers, technology providers, and end users to consult when developing, deploying, and evaluating intelligent AAL technologies.

The paper continues as follows: the Methods section includes the methodology of the scoped literature review; the Results section describes the results of the analysis of the 108 selected articles; and the Discussion section contains the discussion of the review’s findings concerning the RQs and outlines conclusions, limitations, and implications for future work.

## Methods

### Study Type

**Figure 1:**
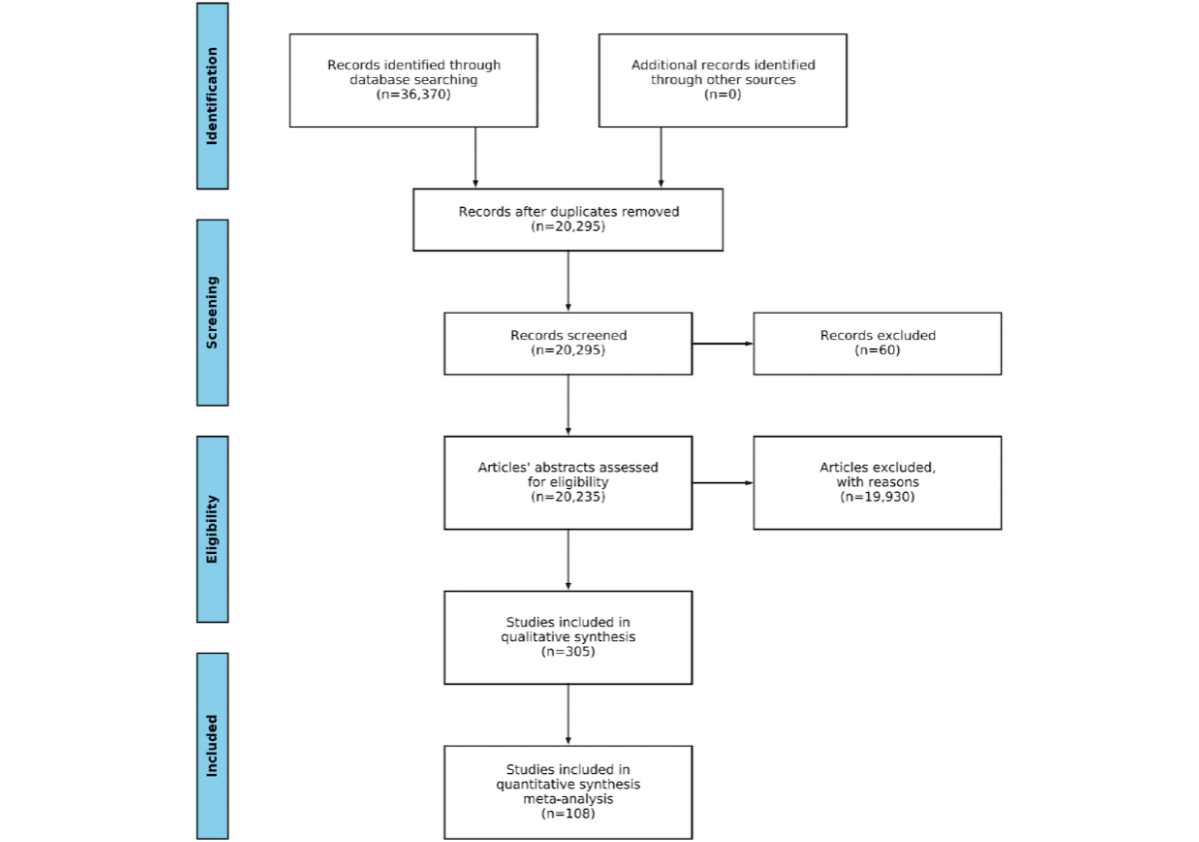
The PRISMA (Preferred Reporting Items for Systematic Reviews and Meta-Analyses) flow of the review process illustrates identification, screening, eligibility, and inclusion of relevant articles.

This paper has been organized as a scoping review, involving the synthesis and analysis of the existing literature to provide a conceptual framework that systematizes and clarifies the specific phenomena—AI models in AAL systems. We identified the articles to be reviewed by conducting a systematic literature search within the IEEE, PubMed, Springer, Elsevier, and MDPI research article databases. The study implemented the PRISMA (Preferred Reporting Items for Systematic Reviews and Meta-Analyses) workflow for systematic reviews [35], as illustrated in Figure 1.

### Identification

During the search, the titles, abstracts, and keywords of the library articles were queried with the search terms structured as in Table 3. The search terms of AAL and AI classification and model categories were mandatory for all articles, and the remaining categories were optional. We ran the search based on categories as properties and used keywords. For this purpose, we used a natural language processing (NLP) toolkit we had developed for automated literature search, screening, and analysis [36]. The toolkit accepts a collection of keywords as input to retrieve potentially relevant articles, combined with the set of properties (or categories) and property groups (as subcategories) to be satisfied by the articles. The input can be expanded with keyword and property synonyms to fine-tune the search and screening process. The details of the toolkit can be found in the article by Zdravevski et al [36].

The search was conducted in July 2022 and included research articles written in English and published between 2010 and 2022. Given the rapid advancements in AI that also influenced the significant growth of technology-supported AAL, we wanted to cover a sufficient research landscape concerning the time frame.

The search process sometimes identified the same article by multiple keywords and phrases from Table 3. For example, the article could describe the use of multiple AI models or classifications. In these situations, we counted the articles multiple times, per each found keyword, and have presented it in Figures 2-13 in the Results section.

**Table 3:** Key terminology for the scoping review’s natural language processing search toolkit.

Category	Criteria	Keywords
Ambient assisted living	Mandatory	*Ambient assisted living, ambient-assisted living, assisted living, active and assisted living,* and *active-assisted living*
Artificial intelligence class	Mandatory	*Supervised learning, unsupervised learning, semi-supervised learning,* and *reinforcement learning*
Artificial intelligence model	Mandatory	*Classification, regression, clustering, dimensionality reduction, association rule learning, instance-based learning, regularization learning, decision tree learning, Bayesian learning, ANN, DL, ensemble learning,* and *natural language processing*
Domain	Optional	*Activity recognition, health monitoring, activity assistance, rehabilitation, therapy, interaction, communication,* and *entertainment*
Technology	Optional	*Mobile technology, mobile device, smartphone, tablet, touch-screen, wearable technology, wearable device, robot, robotic device, ambient sensing, ambient sensors, game, gamification, conversational agent, chatbot, virtual assistant,* and *virtual companion*
Beneficiaries	Optional	*Older adults, frail persons, patients, healthcare staff, caregivers,* and *family*
Concerns	Optional	*Acceptance, adoption, availability, accessibility, privacy, usability, reliability, safety,* and *security*

### Screening

In the screening phase, we evaluated the retrieved articles to assess their relevance to the review based on the independent inclusion and exclusion criteria presented in Textbox 1.

The first 3 authors (MJ, GM, and EZ) manually screened the content of each article independently and coded it to indicate its relevance concerning the inclusion criteria. The inclusions were cross-checked, resolved, and confirmed during regular discussions among the authors.

**Inclusion and exclusion criteria.**

**Inclusion criteria**

- Artificial intelligence (AI) classes and models of ambient assisted living (AAL) applications and platforms, where specific classes and models are explicitly considered and not mentioned without description, analysis, or evaluation.
- Articles contributing to the AI models’ domains to support or assist in specific health-related or daily activities, in line with research question 2.
- Articles demonstrating different AAL technologies that use the models and deliver the automated decisions of AAL systems to end users, as per research question 3.
- Articles describing end users’ concerns regarding the model’s automated decision-making outcomes, according to research question 4.
- The primary end users are older adults, but end users also include other user groups.

**Exclusion criteria**

- Articles containing the search terms, but AAL, AI classes and models, domains, technology, and end users’ concerns were not scrutinized. Thus, they were not relevant to research questions 1-4.
- Literature reviews and surveys on the related topics.

### Extraction

In this phase, we analyzed each included article in detail. We identified and extracted AI classes and models in AAL systems, the models’ target domains and technologies, and the end user’s categories and concerns, where available per article. The extracted information from the articles was kept in a shared spreadsheet to facilitate coding and discussion among the authors. The extracted information included publication venue and date, a summary of the article, AI model or models used including corresponding AI algorithms and tools, the models’ target domains if available, the technology using the models, if any, and information on the end users and their concerns regarding the models if available.

### Analysis

We conducted a manual, thematic analysis of the extracted information during this phase. Our goal was to categorize the AI classes, models, domains, technologies, and concerns for AAL systems. Coded data were the basis to address the review’s RQs. In particular, we grouped articles based on their primary outcomes to guide the analysis as follows: articles that describe AI classes and models of the AAL systems, articles dealing with the models’ domains, articles that present the technologies using the models, and articles with the models’ beneficiaries and use concerns.

We describe the general approach to analyzing the particular article groups.

*The analysis of AI classes and models in AAL * systems concerned identifying and describing the systems’ automated learning and decision-making functionalities, including the particular AI algorithm or tool. *Analysis of AI models’ domains* considered specific application scenarios with supported activities. *Analysis of the AI models’ technologies* through which the automated decisions were generated and communicated to the end users. *Analysis of the models’ concerns* included various end users’ perceptions and dispositions toward the models’ functions and outcomes.

## Results

### Screening Process and Number of Articles

The NLP search toolkit initially identified 36,370 potentially relevant studies (Figure 1). Duplicates were then eliminated, reducing the number to 20,295. The automated screening process further removed 60 articles published before 2010 or for which the title or abstract could not be analyzed owing to parsing errors, unavailability, or other reasons. The NLP toolkit’s advanced functions assessed the eligibility of the remaining 20,235 articles and kept 305 articles. After automated processing, the articles were analyzed in detail according to the inclusion and exclusion criteria. Finally, 108 articles were deemed eligible for the in-depth manual investigation to identify and articulate research results, trends, and implications. The articles are reported in Multimedia Appendix 1 [27,37-142].

We describe the results by responding to the RQs that guided our review.

### Distribution of Relevant Articles and Categories

**Figure 2:**
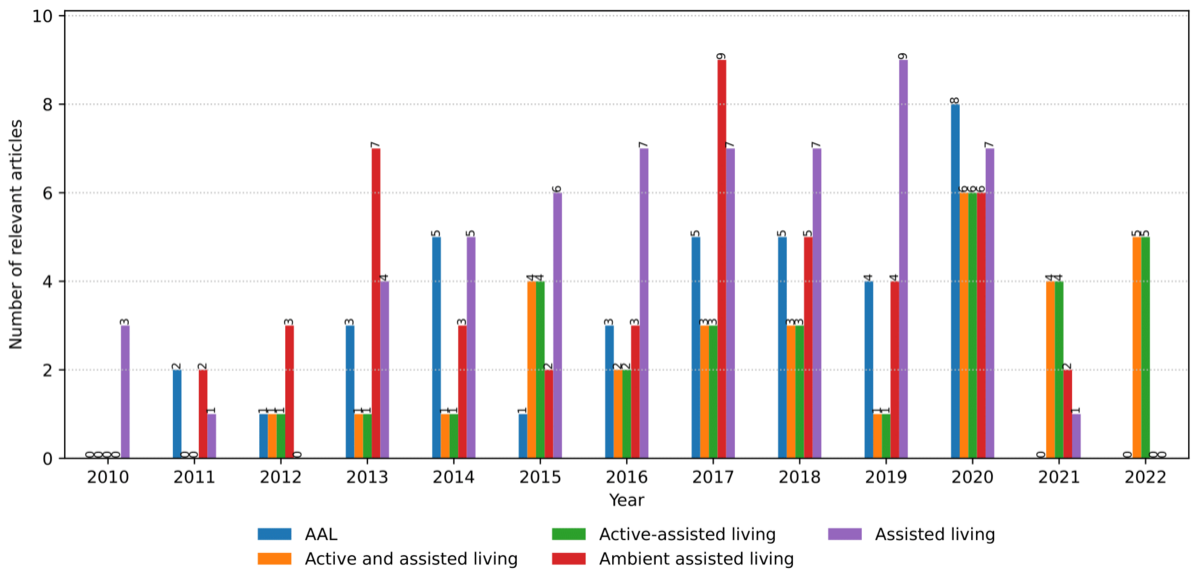
The number of relevant articles concerning ambient assisted living (AAL) with artificial intelligence classes and models per year from January 2010 to July 2022.

Figure 2 illustrates the annual occurrences of the relevant articles containing different AI classes and models. The term “assisted living” has been commonly used in the literature to describe the systems with similar context and purpose of use as per the definition of AAL [1,4]. It outperformed the number of articles in some years (eg, 2019) and was comparable with the AAL in 2018 and 2020. In minor cases, the abbreviation was used solely. In general, there has been a growing trend throughout the search time frame, occasionally decreasing in specific years. The decreases were owing to our search conditions and inclusion criteria. Many articles dealt with AAL without explicit mentions of AI models concerning their application and outcomes.

**Figure 3:**
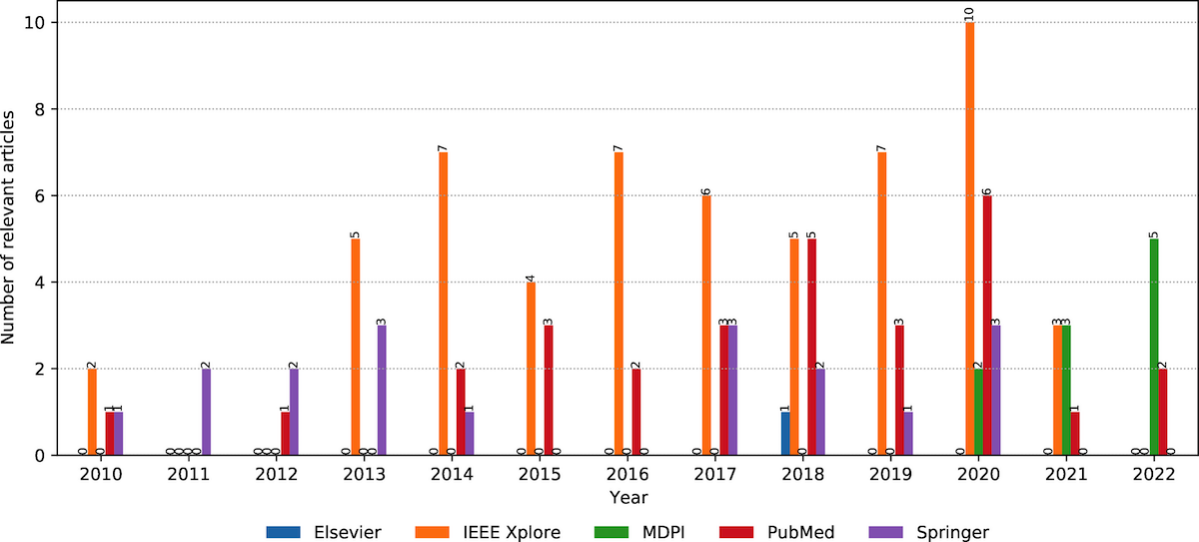
The number of relevant articles per year from January 2010 to July 2022, grouped by the respective digital library.

The combined information on the digital library and publication year of the relevant articles demonstrates that IEEE is a leader, with an increasing trend, reaching a peak in 2020 (Figure 3). This is expected, as the publisher is oriented toward technology, with many venues relevant to AI models and AAL. PubMed follows, dealing more with the end-user aspects of the topics, such as different types of user evaluations. We could notice a growing trend until 2020 and an oscillatory period afterward. The Springer library combines technical articles with user-oriented articles. A smaller number of the relevant articles with an irregular annual trend was found in the Elsevier library, while MDPI published relevant articles from 2020.

**Figure 4:**
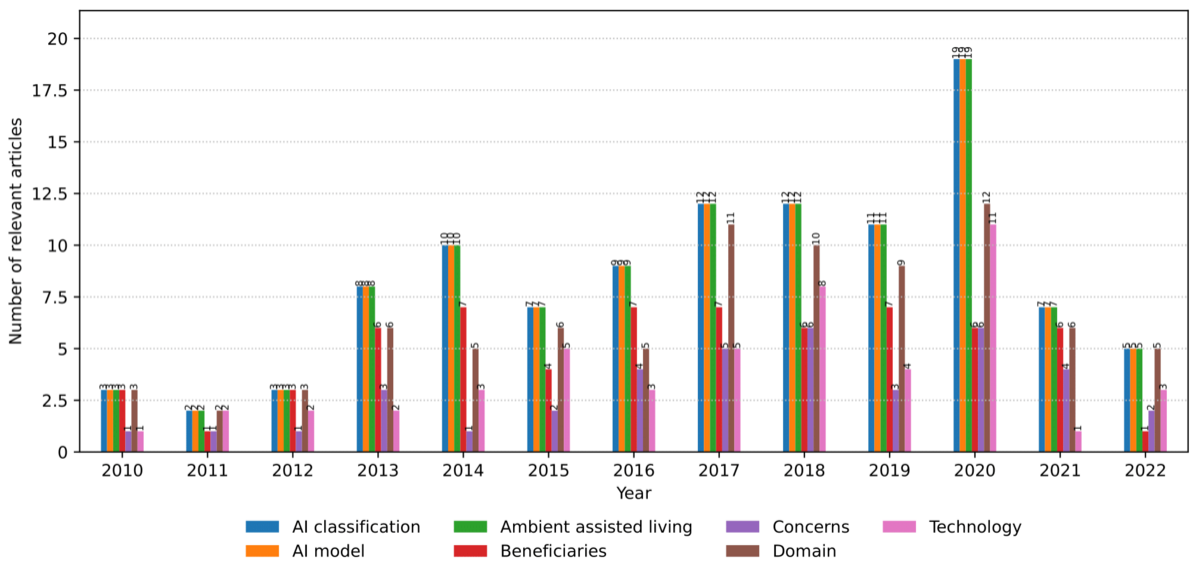
The number of relevant articles for each category per year from January 2010 to July 2022. AI: artificial intelligence.

As the total number of relevant articles increased within the review time frame, the number of articles pertinent to the associated categories changed accordingly (Figure 4). As for the 3 mandatory categories (*AAL*, *AI classification*, and *AI model*), there is a general growing trend up to 2020, with occasional drops in the previous year and a decrease in 2021. As an optional category, the *domain* follows the leading trend but with fewer articles, indicating that sometimes it was not considered (ie, AI models used or tested in a domain-independent way). The *beneficiaries* follow the leading trend but are smaller than the domain, showing that the AI models are sometimes studied without relating to a particular user group or groups. The *beneficiaries* are comparable with the technology in total amount but with annual oscillations due to different types of AI model verifications across relevant articles (ie, deployments and evaluations with or without users). The *concerns* appear in the smallest amount that grows in time and oscillates in some years, showing that relevant articles focused on various aspects of AI models in AAL, beyond and different from users’ concerns (ie, algorithmic accuracy and performance).

### Connections Between and Within Categories

Our analysis revealed overlap between searched categories. We aimed to represent all categories equally while highlighting particular connections as informative (eg, notably higher co-occurrences of instances from distinct or within the categories).

**Figure 5:**
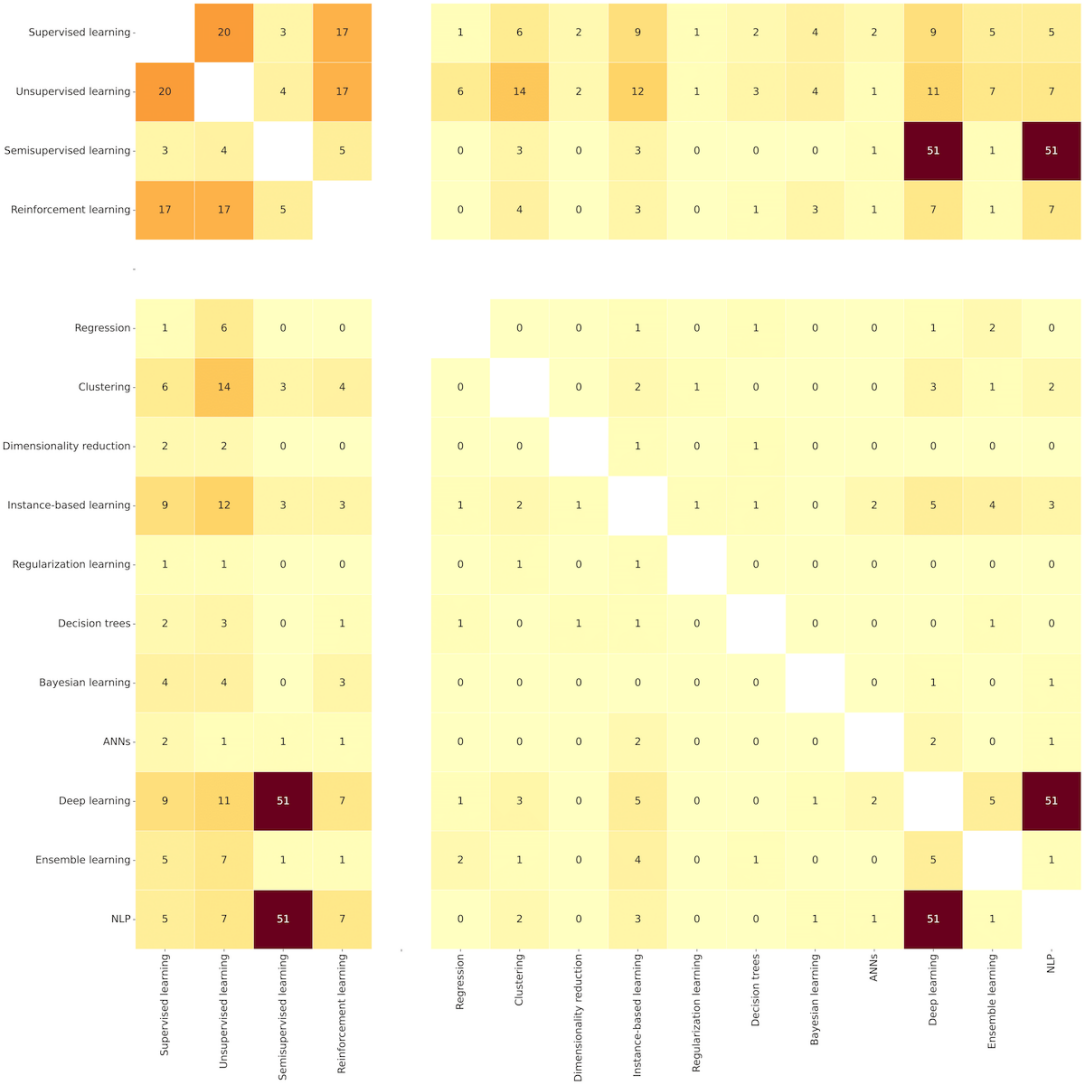
The heat map describing co-occurrences of artificial intelligence classes and models in relevant articles. ANN: artificial neural network; NLP: natural language processing.

Figure 5 shows associations of AI models and the classes they use. Semisupervised learning is a dominant approach for deep learning (DL) and NLP models (51 occurrences). Unsupervised learning appears mainly in clustering (14 occurrences), instance-based learning (12 occurrences), and DL (11 occurrences). Supervised learning prevails for instance-based learning and DL (9 occurrences per model). Finally, reinforcement learning was the occasional approach for DL and NLP (7 occurrences per model).

The study reveals specific synergies within categories. Regarding the classes, 20 articles combined supervised and unsupervised learning. Reinforcement learning was used together with the previous 17 times per class. The studies combined the classes in a sequence or for mutual comparison in solving concrete problems. Concerning the models, we noticed that NLP tasks have been mainly tackled with DL algorithms and tools (51 occurrences).

**Figure 6:**
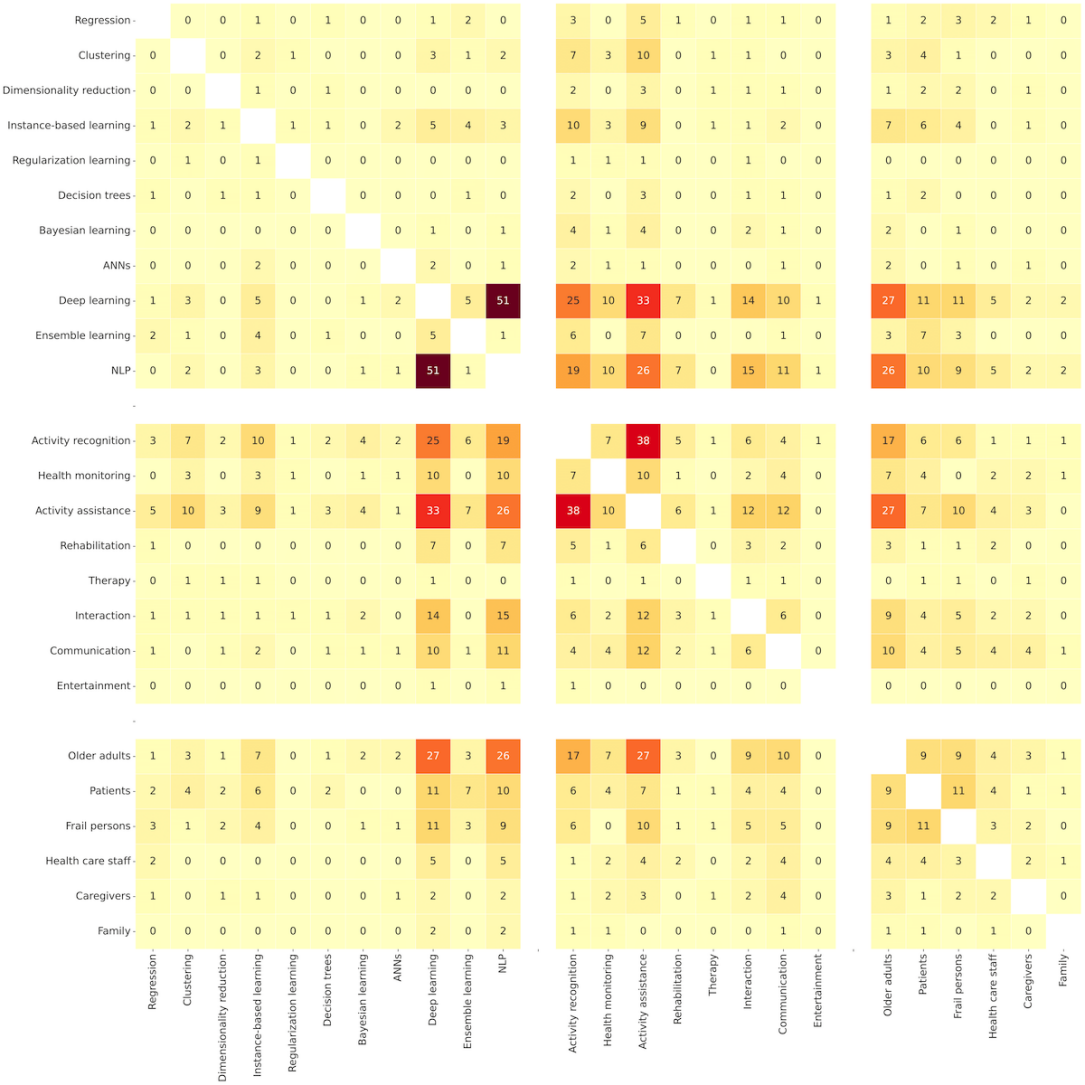
The heat map describing co-occurrences of artificial intelligence models, domains, and beneficiaries in relevant articles. ANN: artificial neural network; NLP: natural language processing.

Figure 6 presents combinations of AI models, domains, and beneficiaries. Activity assistance (33 occurrences), activity recognition (25 occurrences), and interaction (14 occurrences) mainly used DL models. Similarly, and to a smaller extent, NLP models helped with activity assistance (26 occurrences), activity recognition (19 occurrences), interaction (15 occurrences), and communication (10 occurrences).

Combinations of AI models and beneficiaries highlight older adults as the leading users of DL (27 occurrences) and NLP (26 occurrences). Patients and frail persons coexisted with DL models 11 times each.

The coappearance of beneficiaries and domains reveals that activity assistance targeted mainly older adults (27 occurrences), followed by activity recognition (17 occurrences) and communication (10 occurrences).

As for connections within categories, activity recognition is a common form of assistance (38 occurrences), followed by communication (12 occurrences), interaction (12 occurrences), and health monitoring (10 occurrences). Patients and frail persons co-occurred 11 times. Older adults were referred to as frail persons and patients 9 times each, indicating that AI models mainly serve healthy older adult users. Family, caregivers, and health care staff rarely appeared together in these articles.

**Figure 7:**
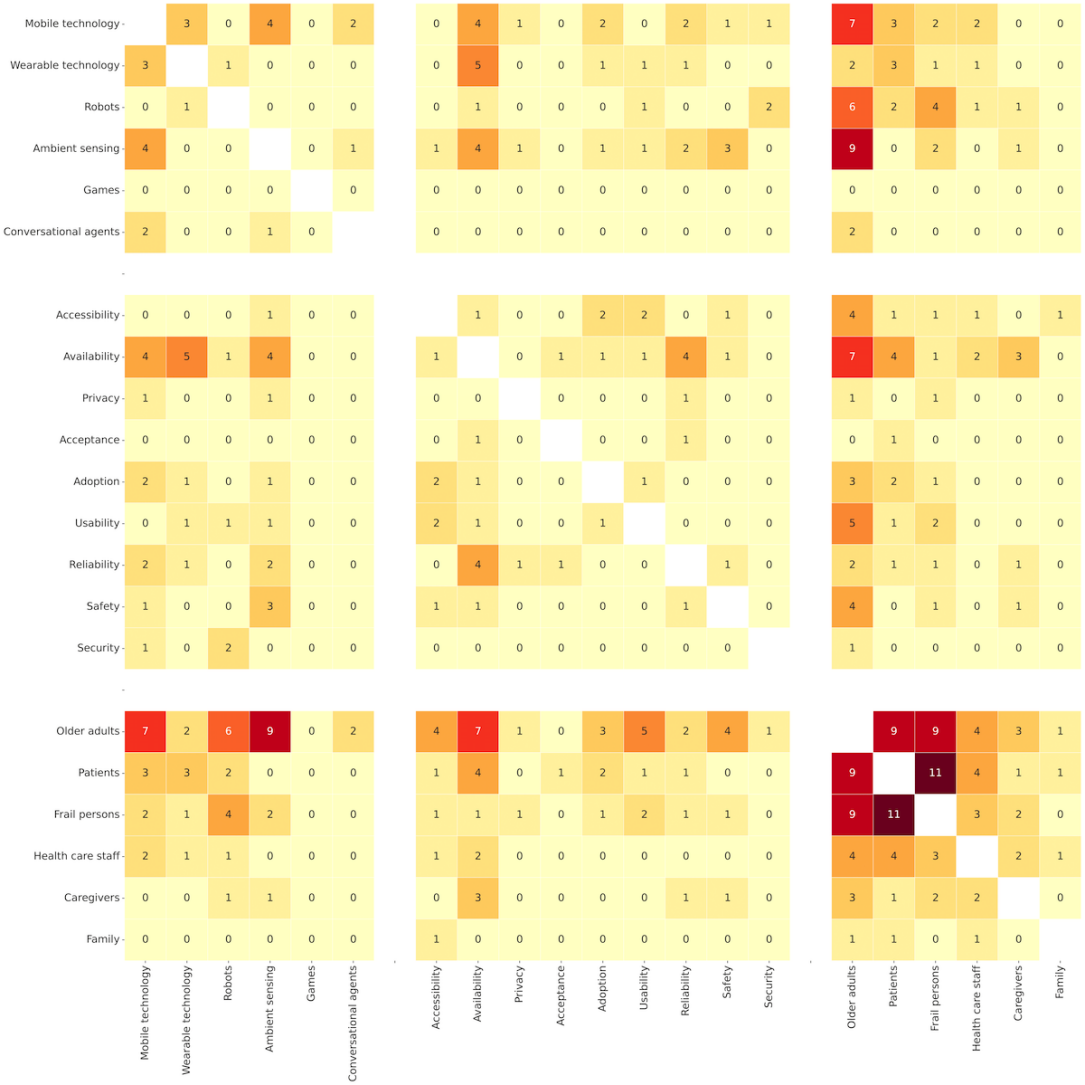
The heat map describing co-occurrences of technology, beneficiaries, and concerns in relevant articles.

Figure 7 shows instances and connections between the technology, beneficiaries, and concerns. Relationships between the nodes from different categories reveal that older adults commonly used ambient sensing technology (9 occurrences), mobile devices (7 occurrences), and robots (6 occurrences). At the same time, their primary concerns were availability (7 occurrences), usability (5 occurrences), and safety and accessibility (4 occurrences per category). Availability is a concern for patients (4 occurrences). Moreover, availability is the primary concern in wearable technology (5 occurrences), along with ambient sensing and mobile technology (4 per category).

Links between the instances within a category indicate occasional use of ambient sensing and wearable technology with mobile devices 4 and 3 times, respectively.

### AI Classes and Models in AAL

**Figure 8:**
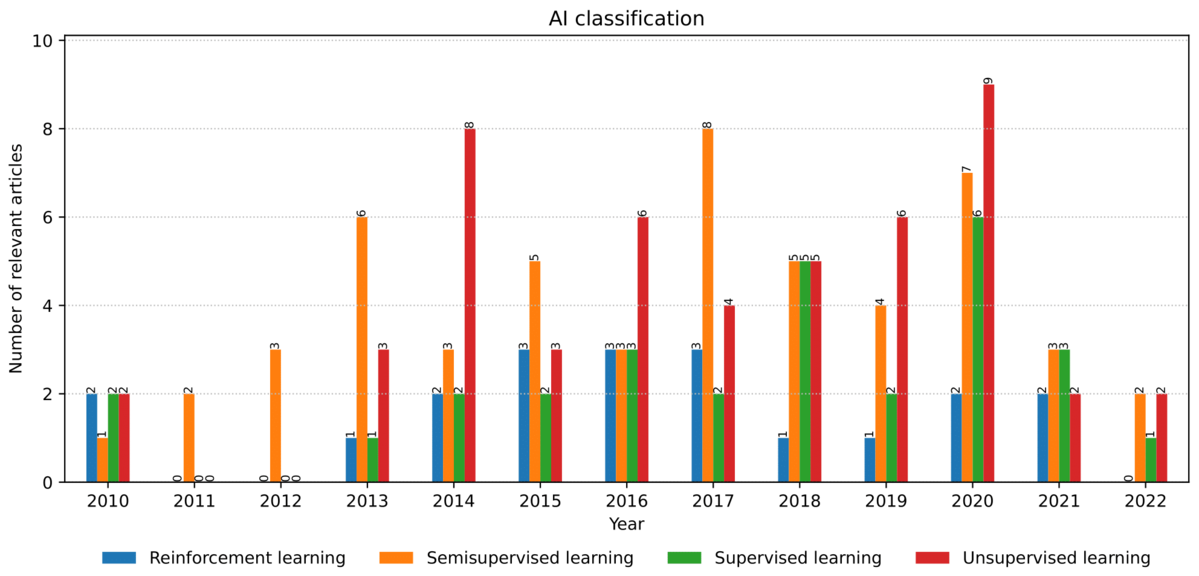
The number and annual distribution of the relevant articles concerning artificial intelligence classes from January 2010 to July 2022.

Concerning the classes, the analysis of relevant articles (Figure 8) showed the highest presence of semisupervised learning (52 occurrences), followed by unsupervised learning (50 occurrences), supervised learning (29 occurrences), and reinforcement learning (20 occurrences).

The distribution of AI classes shows *that semisupervised learning* models had prevailed since the 2010s, with an irregular growing trend until 2017, when they compensated for the lack of a sufficient amount of labeled data for particular inputs. Concrete examples include clustering for physical activity recognition [37], finding relevant input features for improving activity recognition [27], and detecting user-object interactions from sequences of images [143].

The *unsupervised learning* model trend follows the previous category, with a slightly smaller number of appearances. Their use was motivated by a general lack of annotated (or labeled) training data for various activities that early AAL solutions aimed to support [4,8]. Such problems were tackled mainly by either grouping according to shared properties or simplifying input data. The growing trend that followed was caused by the emergence of new health-related domains and activities that solutions were targeting and for which the labeled data did not exist. The examples include the recognition and measurement of everyday activities from unlabeled data [38], clustering to create an ontology of human activities [39], or classification for predicting user movements indoors [40].

*Supervised learning* models showed general growth until 2020. They complemented other approaches (eg, unsupervised and reinforcement learning; Figure 5) for particular user activities for which labeled data existed. They were used in various *classification* tasks within the AAL, such as user reidentification with red green blue depth (RGBD) cameras [41] and ADL recognition using wearable sensors [42].

*Reinforcement learning* models were used from 2010, increasing use until 2015 and reducing use after 2017. They have served as alternatives to data-driven approaches (ie, clustering and regression) by promoting desirable behaviors and eliminating undesirable user behaviors. Hidden Markov models are the most common algorithms in applications, including user activity recognition from appliance consumption data [43] or with multiple Kinect devices [44].

Regarding the models, the study revealed the prevalence of DL (63 occurrences), followed by NLP (54 occurrences); instance-based learning (20 occurrences); clustering (17 occurrences); ensemble learning (12 occurrences); regression (7 occurrences); Bayesian learning, decision tree learning, and dimensionality reduction (4 each); artificial neural network (ANN; 3 occurrences); and regularization learning (2 occurrences).

**Figure 9:**
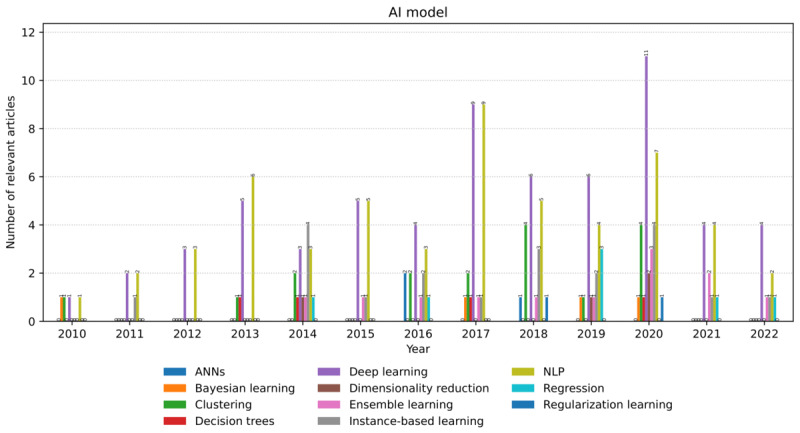
The number and annual distribution of the relevant articles concerning artificial intelligence models from January 2010 to July 2022. ANN: artificial neural network; NLP: natural language processing.

In the following sections, we describe each model according to its prevalence (Figure 9).

*DL* models gained momentum in 2017, expanding their use cases up to date. Their application assumes labeled input data of different structures and semantics are generated at scale. Convolutional neural networks are the most common algorithms used independently or in combination within this model. Examples are activity recognition by transforming data from smartphone sensors into image-based representations [45] or detecting human postures using RGBD cameras [46].

The use of *NLP* models can be divided into 2 stages—earlier applications (up to 2015), which focused on speech recognition and natural language understanding, and later applications, which could also perform dialog management and natural language generation. We can explain this trend with the critical advancements in conversational AI facilitated by the DL algorithms that overlap with our search time frame [34]. For example, the detection of acoustic events (eg, knock, cough, and clap) for older adults in ADL [47] versus conversation with a companion robot [144].

*Instance-based learning* models were used throughout the search period, with an irregular trend and a recent drop from 2020. They used mainly k-nearest neighbor and support vector machine algorithms. The use cases include recognizing physical activity patterns at home with a multiview infrared motion sensing system [48] or detecting ADL from human joint trajectories captured with a depth camera [49].

*Clustering learning* models were used in specific years of the search time frame, mainly unsupervised, as an alternative approach in the absence of labeled data concerning particular use cases. The use cases include predicting a sequence of connected users’ actions in a robotic device [50] or detecting dining-related postures from motion sensor data [51].

*Ensemble learning* models were used starting from 2014. Boosting and random forest are the main algorithms in this model, including physical activity classification from wearable sensors [52] and seizure and fall detection from a smartphone’s accelerometer data [53], respectively.

The remaining models were used to a smaller extent during the search time frame.

*Regression learning* models were mainly linear regression, such as real-time energy expenditure estimation when walking with loads and on inclines assisted by an ankle exoskeleton [54] or ADL recognition from hand grasps using electroencephalography [55]. *Bayesian learning* models were applied to classification problems such as ADL recognition (ie, detection and classification) using data collected from wearable motion sensors [42]. *Decision tree* and *dimensionality reduction* models were used for classification tasks. The respective examples classify physical activities based on step counts [56] or Wi-Fi and wearables’ data [57]. ANNs were applied to classification problems as a predecessor of DL models, such as activity recognition in safety-critical environments (eg, fall detection) [58]. Finally, *regularization learning* models were applied to regression tasks, such as selecting predictive input features for person identification with RGBD cameras [41].

Although they were mentioned in some articles in the context of previous, relevant, or future work, our analysis did not reveal the examples of *association rule learning* models’ algorithms in AAL.

### Domains of AI Models

**Figure 10:**
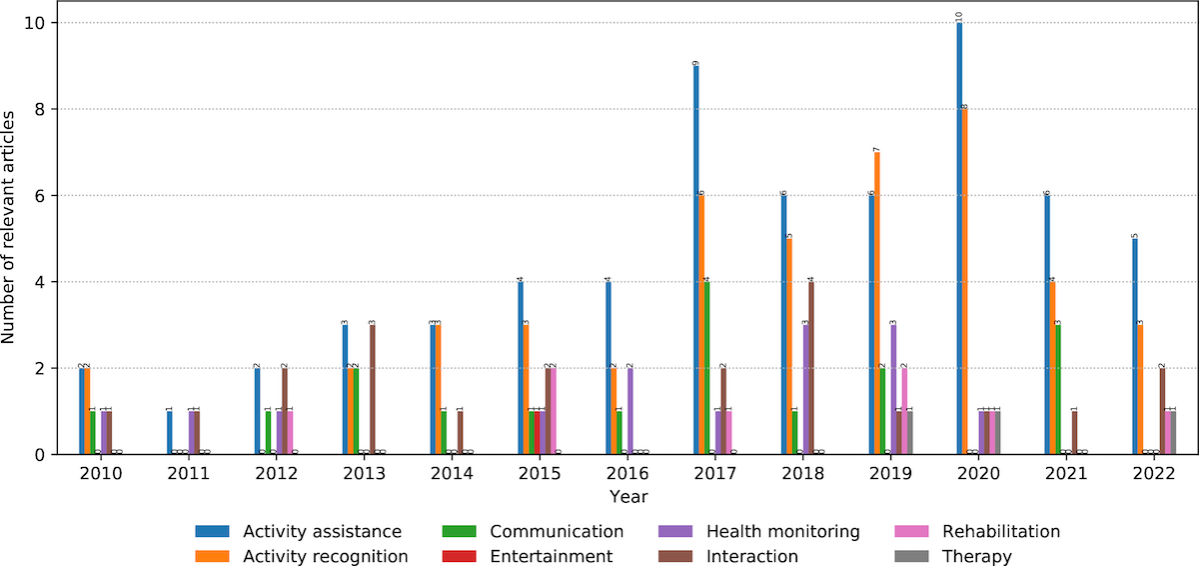
The number and annual distribution of relevant articles concerning the artificial intelligence models’ domains from January 2010 to July 2022.

AI models were applied in multiple domains, per domain, or in combination (Figure 10). The most popular domains found were *activity assistance* (61 occurrences) and *activity recognition* (45 occurrences). The domains showed a growing trend until 2020, with periodic oscillations during the time frame. As indicated earlier, they were mainly interconnected in previous studies (38 occurrences; Figure 6). Activity assistance has been a significant target in AAL and assistive technologies in general. Mobility is a common assisted activity, such as a robotic walker for mobility in older adults [59] or smart glasses helping visually impaired users navigate physical spaces [60]. Human activity recognition (HAR) is a commonly used term to describe the recognition of various physical activities. These activities are usually classified into ADL (health focused) and instrumental ADL (IADL; well-being focused), indicating that intelligent AAL systems support health and quality of life. An essential challenge in activity recognition is predicting long-term behavior [61]. Similarly, some studies dealt with the problem of multisensor data fusion in a robotic walker for indoor assistance [62].

*Interaction* (21 occurrences) referred to the use of different AAL systems, whereas *communication* (17 occurrences) was mainly considered from a technical perspective (eg, communicating between sensors, servers, and cloud-based systems). The former examples include interacting with an innovative home platform for facial emotion recognition [46] or a medication delivery application [63]. The latter is a distributed multimedia system for patient data capture [64] or digital footprint applications for the activity prediction of assisted users [65].

*Health monitoring* (14 occurrences) was commonly referred to as observing users’ vital signs to detect changes in health conditions and emergencies, such as health-related data collection for users at their homes [64] or in-home gate analysis using radar sensors [61].

Rehabilitation (8 occurrences), therapy (3 occurrences), and entertainment (1 occurrences) received less attention from the research community in the search time frame. A rehabilitation example is a home system suggesting medications and exercises during fall recovery [66].

### Technologies Using AI Models

**Figure 11:**
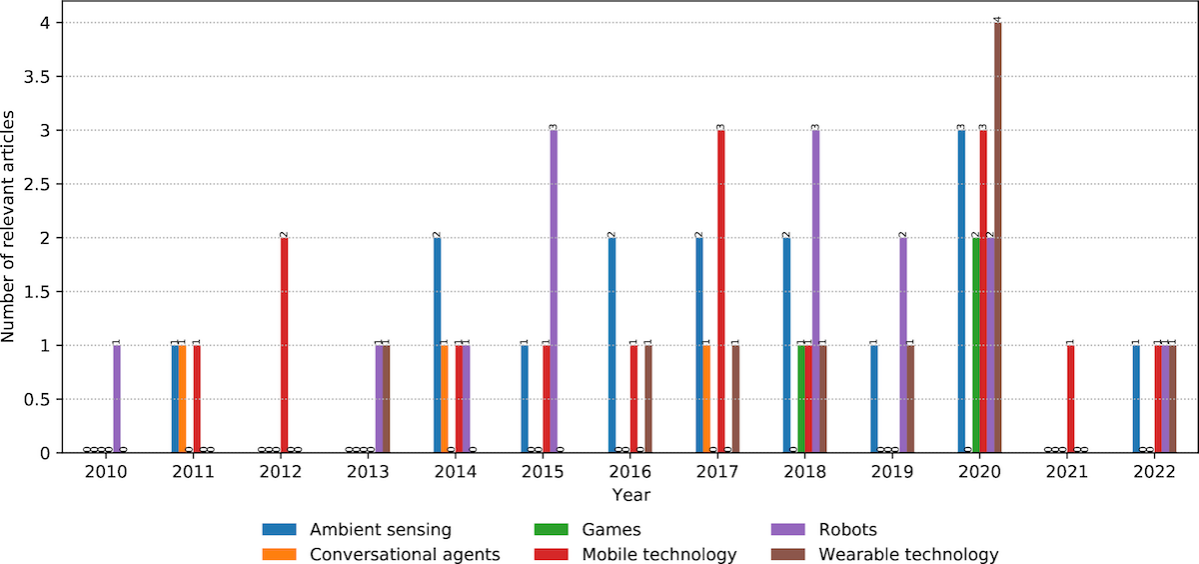
The number and annual distribution of relevant articles concerning the artificial intelligence models’ technology from January 2010 to July 2022.

*Ambient sensing* and *mobile technology* (15 occurrences per category) prevail in AAL (Figure 11). It is an umbrella term that connotes various sensors that measure the parameters of the observed environment (or ambient environment) to detect and analyze user behavior. In this respect, studies used a particular sensor or combined multiple sensors. In the former case, vision [49] and radar [67] sensors were used for recognizing activities and measuring vital signs, respectively. In the latter case, studies merged signals from various sensors for energy efficiency and improved accuracy and performance (known as sensor fusion). For example, activity recognition combined depth image sequences and audio data [68].

*Mobile technologies* exposed 2 typical roles. A *passive role* in using their embedded sensors and providing a user interface for measuring the conditions in the users’ environments or the state of their behaviors [53]. An *active role* in promoting healthy habits and behaviors among users for a positive lifestyle change by suggesting activities [65].

*Robotic technology* (14 occurrences) was used during the time frame, with an irregular trend. Robots fit well with the AAL paradigm, as they replicate human abilities and characteristics, but the cost of development and deployment may influence their use. In line with related work, we noticed their *assistive* and *companionship* purposes. The former concerns upper-limb gesture recognition to help users with ADL [69]. The latter is demonstrated by interacting with older adults to prevent social isolation and mediating between the older adult, the environment, and the AAL system [144].

*Wearable technology* was used less frequently than previously (10 occurrences). On the one hand, it can introduce a certain level of intrusiveness compared with ambient sensors when used independently. On the other hand, it is available through mobile devices (eg, smartwatches and bracelets), and the study identified 3 overlaps (Figure 7). The study by Slade et al [54] used wearable sensors attached to users’ ankles to estimate energy consumption when walking. Another example is activity recognition, which accounts for measurement uncertainty in wearable sensors [42].

*Conversational* and *gaming* technologies have 3 occurrences each. The conversation example is a social robot that conducts simplified small-talk dialogs with users [144]. Overall, the dialogs were rare compared with the many occurrences of NLP models used for speech and text recognition. Games were mentioned as the use of gaming technologies (eg, Kinect RGBD camera) that recognized human activity [145].

### Beneficiaries of AI Models

**Figure 12:**
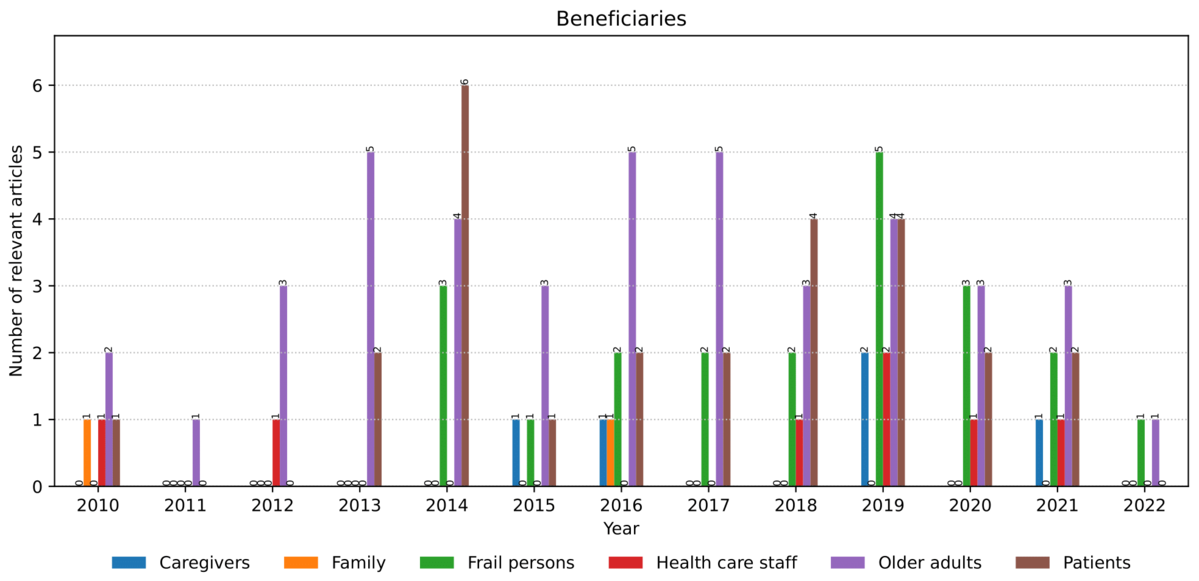
The number and annual distribution of relevant articles concerning the artificial intelligence models’ beneficiaries from January 2010 to July 2022.

*Older adults* are the primary beneficiaries of intelligent AAL systems (42 occurrences; Figure 12). The shared elements emerging from different AAL systems using AI models for this target group include HAR and measuring vital signs. Accordingly, the study described by Saeed et al [70] used radar sensors’ data to infer the activities of community-dwelling older adults, while research by Ejupi et al [71] detected falls by analyzing accelerometer and barometric pressure sensor data.

*Patients* (n=26) were persons with health declines who underwent different medical treatments. Examples include activity prediction for fall prevention of patients at risk [44] and diagnosing clinical abnormalities of patients using multiple vital signs (eg, heart rate, blood pressure, and respiratory rate) [72]. The overlap with older adults (9 times; Figure 6) indicates that for most older people, the purpose of AAL systems was more assistive and aimed toward health promotion rather than therapy.

*Frail persons* (n=21) were in a specific state of vulnerability with increased risks of falling or disability. The AAL support for these beneficiaries was manifested in diagnosing various health declines. Examples include diagnosing Alzheimer disease from magnetic resonance images [73] and detecting emergencies with users’ mobility [54].

Health care staff (n=7), caregivers (n=5), and family (n=2) were considerably less present than previously. Owing to the AAL technology used, they appeared as beneficiaries concerning more efficient and effective caregiving. For example, supporting medical staff in monitoring patients at home [66] and notifying physicians and families if patient conditions decline[64].

### Concerns in AI Models

**Figure 13:**
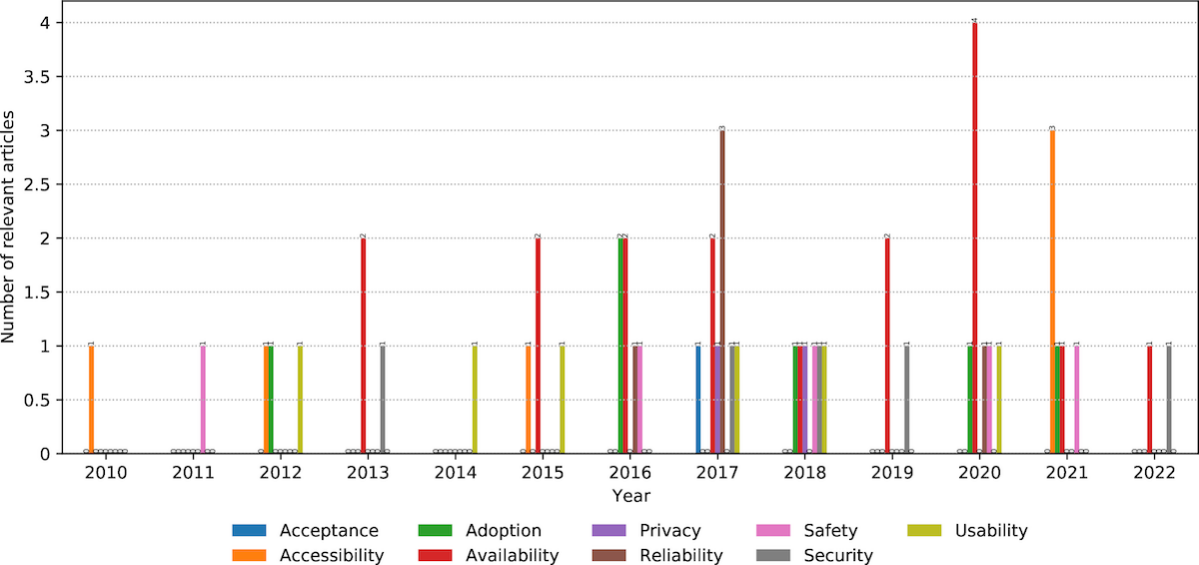
The number and annual distribution of relevant articles regarding the beneficiaries’ concerns from January 2010 to July 2022.

*Availability* of intelligent AAL systems was the primary user concern (17 occurrences; Figure 13). It was mentioned mainly concerning a particular technology. For example, beneficiaries preferred off-the-shelf technologies, such as mobile devices because of their availability regarding services they can offer and cost [53]. Conversely, the availability of particular devices, such as exoskeletons [54] or multiple Kinect devices [145], was highlighted as a potential barrier to their use.

*Accessibility*, *adoption*, and *usability* appeared 6 times each. The accessibility of the AAL systems’ services reflects the convenience of reaching them, such as the functions of an Internet of Things device that generates user profiles from their activities [74]. The adoption referred to a more sustained and stable use and integration of the introduced technology into the beneficiaries’ routine, such as the use of widely adopted technologies (ie, smartphones) for HAR [45]. Usability was described as the ease of use of various AAL systems, including the percentage of successful task completion when using a medication management application [63].

*Reliability*, *safety*, and *security* had 5 occurrences each. Reliability describes the reliability levels of the AI models’ outcomes from the beneficiaries’ perspective, such as the perceived accuracy of activity trackers [56]. Safety is a requirement for AAL applications to prevent any harm to their users, such as detecting abnormal human behaviors to avoid dangerous situations [75]. Security refers to protecting users from external threats when using AAL technology; for example, when using users’ appliance consumption data to infer their activities [43].

*Privacy* (2 occurrences) and *acceptance* (1 occurrence) received the least attention. Privacy manifested as the need to protect beneficiaries’ data during collection, analysis, and use by the AAL system, such as protecting persons’ identities [41]. Acceptance emerged as a desired quality of the AAL technology that facilitates the attitude, such as the unobtrusiveness of radar-based sensors for patient monitoring [61].

## Discussion

### Overview

This section summarizes the results of the scoping review. The AI models are key drivers of AAL systems. In this respect, this study clarifies their role and significance over the previous decade by considering domains, technologies, and end users. At the same time, it highlights critical user concerns to identify gaps that require further research.

The overall goal was to provide an overview and synthesis of the research on AI classes and models in AAL (RQ1), domains in which they were applied (RQ2), technologies that used them (RQ3), and their beneficiaries along with use concerns (RQ4).

The following section discusses the principal findings concerning the evolution of the AI models and related categories and implications for different stakeholder groups, including well-being and health care, technology, and research.

### Principal Findings

#### Evolution of Categories in AAL

The time frame has seen a variety of AI *model* contributions that target a range of *domains*, *technologies*, and *beneficiaries*. Semisupervised and unsupervised learning classes dominate the intelligent AAL landscape. Their prevalence is because of an increase in the variety of the health and living domain and the gradual appearance of labeled input data describing related ADL and IADL the learning aimed to support [37,38,143]. Supervised approaches have been used for classification tasks [41].

DL and NLP models have been mainly used throughout the search time frame. DL models combined neural network–based algorithms such as convolutional neural network and recurrent neural network [146]. These algorithms can be both supervised and unsupervised but were rarely considered explicitly in relevant articles. However, an in-depth manual article analysis showed that they were mainly supervised. These algorithms deal with multidimensional input data from heterogeneous sources. The data describe various human activities to support or infer health conditions [45,46]. In the first half of the frame, NLP models mainly recognized users’ spoken input [47]. During the second half, they enabled conversations with users [144]. Models using reference examples (ie, instance-based learning) and clustering were used for classification tasks [48-51]. Ensemble approaches, by definition, combine separate models to compensate for individual drawbacks [52,53] and were used later in the time frame (from 2014). However, other approaches were notably less frequently used.

Activity assistance and recognition were the leading domains, with a generally growing trend. In most cases, the activity assistance assumed recognition (38/61, 62% occurrences), while the remaining instances focused on specific activities known in advance. A range of ADL and IADL were supported, where different indoor and outdoor mobility (ie, walking, physical exercise, and transportation) prevailed [59,60]. The interaction referred to the systems as seen by their end users [63]. Communication denoted internal interrelations among AAL system components [64]. Health monitoring concentrated on deviations in vital functions and detection of abnormal behaviors [61].

Ambient sensing and mobile technology are mainly used in AAL. Sensing uses different sensors to detect available signals that carry specific information on user behavior [68]. Mobile technologies are convenient to use (ie, market availability, affordability, and wide adoption) at the application level as lifestyle applications for health and well-being [65] and at the device level as a platform with integrated sensors [53]. Robots appeared as either assistive devices helping users carry out their activities [69] or companions for pleasurable activities [144].

The study found notably fewer wearables, followed by conversational and gaming technologies.

Older adults were the primary beneficiaries of AI models in AAL within the search time frame [70]. Patients coexisted with older adults 9 of 26 times or 35% of found cases (Figure 6). This shows that other ages benefited from AI models [44,72]. Frail persons were less present and coappeared with older adults in 43% (9/12) of instances. Health care staff, caregivers, and families were underrepresented compared with the former and occasionally mentioned.

Availability prevails as a beneficiary concern. In general, off-the-shelf, affordable technology [53] is preferred compared with more expensive equipment concerning cost and deployment [54]. The remaining concerns had fewer cases.

#### Implications for Health Care and Well-being

On the basis of our observations concerning domains, beneficiaries, and concerns, we identified gaps in the existing literature and articulated the following directions for future work:

- Collaborative decision-making—current AAL systems make their decisions autonomously, driven by the models’ algorithms and input data. The involvement of expert users (ie, health care staff and caregivers) in the decision-making process can improve its accuracy, facilitate automated learning about users, and reduce the burden on health care professionals.
- Augmenting caregivers and recipients—by definition, the AAL occurs outside health care facilities. In such a scenario, consideration of caregiving and caretaking is critical for adherence to health care services that should address the participants' concerns. Active participation of these beneficiaries is crucial for a successful digital health care intervention, from their AI model comprehension to a particular technology design and deployment.
- AAL interventions—studies included various technologies and platforms to support independent living. Our analysis did not reveal knowledge exchange among studies concerning their results and experiences. Technology-supported health care interventions have been designed for various medical domains. Systematized knowledge of models, domains, technologies, and beneficiaries can guide AAL interventions tailored to specific health care requirements. Such knowledge can reinforce best practices and mitigate potential risks.
- Regulations and compliance—at present, AAL design and deployment space are not regulated, nor is their compliance acknowledged and endorsed by regulatory authorities globally. AAL systems must comply with regulations at both national and international levels. This is crucial for their implementation in medical practice and general adoption. To meet this need, we advocate for a repository of evaluation methods and design guidelines that would support compliance and provide a clear view of how to incorporate critical aspects during AAL system design.

#### Implications for Technology

The analysis of the models, technologies, and concerns revealed unsolved matters that require more attention, including the following:

- Transparency and privacy—AI models, by their very nature, need, produce, and process large amounts of various user-related data, from intensive data collection and analysis to delivering their decisions as personalized recommendations to users. First, the technology should be transparent on why and how user data are collected, analyzed, and used. Second, it should respect a user’s right to control their private data and communications and that they are free from intrusion. Satisfying these user needs is critical for building trust in AAL systems.
- Integration with health care services—AAL systems are usually built and deployed as stand-alone platforms, independent of institutional health care systems. Connecting with existing medical technological infrastructure and digital services can increase the efficiency and effectiveness of health care provision. These benefits are mutual. AI models can be fed with existing user medical records and procedures for improved decision-making. In turn, medical actors could be timely informed of emergencies or changes in users’ behaviors that are difficult to observe in clinical settings.
- Inclusive AAL—AI models focus on individual users as a user-system relation. Group dynamics are not supported, such as user-system–physician relations or forming peer groups of similar users. Future intelligent AAL systems should equally engage and moderate multiple beneficiaries: patients, families, caregivers, and health care staff. This also represents a general implication for health care systems.

#### Implications for Research

Looking at the results as RQs’ responses, following research directions emerged:

- Explainable decision-making—as capabilities of AI models increase, the absence of explanations behind automated behaviors raises uncertainty with users due to a lack of understanding of how specific decisions are made [147]. The explanatory behavior of the models can ingrain positive behaviors to maintain a healthy lifestyle [148]. Thus, a general requirement for future AI models is the provision of explanations understandable to beneficiaries without background or knowledge in AI (ie, nonexperts).
- Evaluation techniques—studies proposed evaluation techniques that could be broadly categorized into functional (ie, technical) and nonfunctional (ie, medical and usability). They used existing instruments to measure AI models’ algorithms (accuracy and performance) or medical and user-related outcomes (standard scales for particular medical conditions, interviews, and questionnaires). Moreover, they focused on a single measure or several measures from the same category. To obtain a clear and valid assessment of the effectiveness and efficiency of the AI models used in AAL, we need a more comprehensive and coherent set of cross-category evaluation metrics to be proposed and verified in practice.
- Design recommendations—the discovery of design guidelines from relevant articles depends on how they are described. In the analysis phase, the identification and extraction of guidelines were not straightforward. The design contributions were mainly presented as suggestions derived from the studies conducted. Other forms included development and deployment practices concerning specific models, domains, and technologies. These contributions are difficult to apply and reproduce, being a barrier to their uptake. Standard reporting procedures and knowledge bases could help address this issue and provide actionable guidelines to interested communities. Several independent studies will be needed to implement and validate the guidelines.

### Limitations

We acknowledge that the proposed AI and machine learning (ML) class and model categorization, which served as a basis for our search, is not comprehensive, exhaustive, or exclusive. Although there are other taxonomies, our goal was to highlight the underlying mechanisms of these classes and models for the review to provide a proper understanding of their roles in AAL systems.

Moreover, the categories and associated keywords may have limited the search results. Thus, we included common synonyms found in the literature as keywords to capture more results at the cost of more nonrelevant articles. Still, we may have missed relevant materials using other terms or not using searched keywords explicitly.

Another limitation of our study was the necessity of setting a time frame for the articles included in the review. We chose to cover work by early reviews of AAL systems and advancements in AI learning algorithms. However, as with any date restriction, there is a risk of not considering potentially relevant work.

A further limitation concerns manual extraction and categorization of retrieved articles (for inclusion), which may introduce a subjective perception of coders. The risk was addressed by cross-analysis and discussion of each other’s results for agreement. Relatedly, the findings on prevalence or trend may primarily represent the researchers’ interest but not an objective sampling of all the stakeholders’ perspectives, including that of the users.

Finally, this study considered 5 digital libraries, among others. Considering the size, coverage, and diversity of digital libraries regarding RQs, we believe that the obtained results sufficiently respond to them.

### Comparison With Prior Work

In comparison with relevant work, we focus on previous reviews and metareviews on related topics and comparative studies, giving preference to AI models. The reviews’, metareviews’, and studies’ scope was generally more constrained than ours.

The meta-review presented by Climent-Perez et al [16] examined video-based lifelogging technologies for AAL in older adults. Lifelogging assumes recording personal data of a user’s daily life. It produces a data set as computational knowledge about a person (also known as quantified self) that could be used for different purposes, such as detecting emergencies and predicting user behavior. The target model was DL, domain HAR, and technology RGBD sensing devices. This study articulates ethical implications for these applications.

The review by Singh et al [149] analyzed existing fall-detection systems through the implementation of existing sensor technologies. It provides a descriptive framework to help choose appropriate sensors for particular deployment scenarios and locations. The main areas for technical improvement were unobtrusiveness, installation costs, and power requirements.

A survey by Demrozi et al [150] discussed ML and DL algorithms for sensor-based HAR of older adults concerning their accuracy and quantity (coverage of recognized activities). ML models require less data and computational resources, whereas DL models better recognize complex activities.

A review of mobile apps for dementia [151] showed that caregivers were the primary users, and the app content mainly provided information on dementia. The barrier to the availability of these apps is a lack of navigating the app marketplace and quality metrics for their dementia information.

A review of DL techniques used in smartphones and wearable sensor-based HAR systems [152] demonstrated that DL techniques outperform other ML ones. However, they were verified on preexisting data sets, not the data acquired in real time.

An in-depth analysis of DL algorithms for HAR using mobile and wearable sensor networks [146] raised the need for higher computational resources in mobile and wearable devices to enable web-based and real-time decision-making.

A more comprehensive review of assistive technologies for older adults classified technologies into clusters, such as general information and communication technology (eg, computer and internet applications), robotics, telemedicine, sensor technology, medication management applications, and video games [17].

A study analyzed randomized controlled trials on the effectiveness of assistive technology for memory support in people with dementia [153]. Measured outcomes included ADL, level of dependency, clinical and care-related outcomes, and perceived quality of life and well-being. The evidence was mixed and inconsistent and drew no generalized conclusions.

Another review investigated mobile health interventions for adults who had experienced stroke [154]. The interventions targeted different patient functions, mostly upper-extremity function, functional mobility, and language and speech skills. However, they were mainly preliminary, focused on technology development up to pilot testing, and lacked evidence from large-scale trials.

Off-the-shelf voice assistants were used by persons with motor, linguistic, and cognitive disabilities [155]. Although these systems are widespread, inexpensive, and nonstigmatizing compared with other assistive technologies, participants’ performances depended on their level of cognitive and linguistic skills.

A comparative study of different ML algorithms for HAR [156] used existing data sets and indicated that sensor-based techniques were preferred over vision-based techniques because they better preserve user privacy. A similar study [157] examined particular algorithms, namely decision tree, k-nearest neighbor, support vector machines, naïve Bayes, linear discriminant analysis, and ensemble learning, in recognizing specific ADL (meal preparation, eating, housekeeping, etc). In general, the algorithms performed equally well on the chosen data set.

### Conclusions

We have described a scoping review based on systematic search and analysis, which identified research trends concerning AI models, domains, technologies, and beneficiaries along with their concerns. The AI models, domains, technologies, beneficiaries, and concerns extracted from the literature represent a knowledge base that can be consulted and used when developing and deploying AI-infused AAL systems. Its findings can (1) inform end users, health care professionals, and caregivers on available technologies and their target medical domains; (2) guide health care providers and engineers in implementing and deploying these technologies; and (3) help end users understand the benefits and trade-offs of the technologies.

Research activity has increased awareness of AI models in AAL and revealed gaps in the field. Further work is needed in making AAL systems more efficient, effective, and user friendly. In particular, hybrid physician-model decision-making, the inclusion of caregivers by technology design, and compliance with health-related regulations will lead to the uptake of AAL by a society. Moreover, improving transparency and privacy, integration with legacy systems, and the equal inclusion of different beneficiaries will improve the acceptance and availability of AAL systems. Finally, efforts to explain automated decision-making, adopt standard evaluation metrics, and verify design guidelines will recognize different AAL approaches to ensure them in digital health care.

## Appendix

Multimedia Appendix 1 The list of the relevant articles retrieved from the 5 digital libraries.

## Abbreviations

AAL: ambient assisted living
ADL: activities of daily living
AI: artificial intelligence
DL: deep learning
HAR: human activity recognition
IADL: instrumental activities of daily living
ML: machine learning
NLP: natural language processing
PRISMA: Preferred Reporting Items for Systematic Reviews and Meta-Analyses
RGBD: red green blue depth
RQ: research question
