# Correction: Ambient Assisted Living: Scoping Review of Artificial Intelligence Models, Domains, Technology, and Concerns

**DOI:** 10.2196/45081

**Published:** 2022-12-20

**Authors:** Mladjan Jovanovic, Goran Mitrov, Eftim Zdravevski, Petre Lameski, Sara Colantonio, Martin Kampel, Hilda Tellioglu, Francisco Florez-Revuelta

**Affiliations:** 1 Department of Computer Science, Singidunum University Belgrade Serbia; 2 Faculty of Computer Science and Engineering, University Saints Cyril and Methodius Skopje North Macedonia; 3 Signals & Images Lab, Institute of Information Science and Technologies, National Research Council of Italy Pisa Italy; 4 Faculty of Informatics, Vienna University of Technology Vienna Austria; 5 Department of Computing Technology, University of Alicante Alicante Spain

In “Ambient Assisted Living: Scoping Review of Artificial Intelligence Models, Domains, Technology, and Concerns” (J Med Internet Res 2022;24(11):e36553) the authors noted one correction.

Under “Acknowledgments”, the sentence:

This work was part of and supported by GoodBrother, COST Action 19121—Network on Privacy-Aware Audio- and Video-Based Applications for Active and Assisted Living.

has been replaced by:

This publication is based upon work from COST Action GoodBrother—Network on Privacy-Aware Audio- and Video-Based Applications for Active and Assisted Living (CA19121), supported by COST (European Cooperation in Science and Technology).

The correction will appear in the online version of the paper on the JMIR Publications website on December 20, 2022 together with the publication of this correction notice. Because this was made after submission to PubMed, PubMed Central, and other full-text repositories, the corrected article has also been resubmitted to those repositories.

